# Genomic variation among populations of threatened coral: Acropora cervicornis

**DOI:** 10.1186/s12864-016-2583-8

**Published:** 2016-04-13

**Authors:** C. Drury, K. E. Dale, J. M. Panlilio, S. V. Miller, D. Lirman, E. A. Larson, E. Bartels, D. L. Crawford, M. F. Oleksiak

**Affiliations:** Rosenstiel School of Marine and Atmospheric Science, University of Miami, 4600 Rickenbacker Causeway, Miami, FL 33149 USA; Nova Southeastern University Oceanographic Center, 8000 N Ocean Drive, Dania Beach, FL 33004 USA; Center for Coral Reef Research, Mote Marine Laboratory, 24244 Overseas Highway, Summerland Key, FL 33042 USA

**Keywords:** Genotyping by sequencing, Coral reefs, Population genomics, Restoration genetics, Florida reef tract

## Abstract

**Background:**

*Acropora cervicornis*, a threatened, keystone reef-building coral has undergone severe declines (>90 %) throughout the Caribbean. These declines could reduce genetic variation and thus hamper the species’ ability to adapt. Active restoration strategies are a common conservation approach to mitigate species' declines and require genetic data on surviving populations to efficiently respond to declines while maintaining the genetic diversity needed to adapt to changing conditions. To evaluate active restoration strategies for the staghorn coral, the genetic diversity of *A. cervicornis* within and among populations was assessed in 77 individuals collected from 68 locations along the Florida Reef Tract (FRT) and in the Dominican Republic.

**Results:**

Genotyping by Sequencing (GBS) identified 4,764 single nucleotide polymorphisms (SNPs). Pairwise nucleotide differences (π) within a population are large (~37 %) and similar to π across all individuals. This high level of genetic diversity along the FRT is similar to the diversity within a small, isolated reef. Much of the genetic diversity (>90 %) exists within a population, yet GBS analysis shows significant variation along the FRT, including 300 SNPs with significant F_ST_ values and significant divergence relative to distance. There are also significant differences in SNP allele frequencies over small spatial scales, exemplified by the large F_ST_ values among corals collected within Miami-Dade county.

**Conclusions:**

Large standing diversity was found within each population even after recent declines in abundance, including significant, potentially adaptive divergence over short distances. The data here inform conservation and management actions by uncovering population structure and high levels of diversity maintained within coral collections among sites previously shown to have little genetic divergence. More broadly, this approach demonstrates the power of GBS to resolve differences among individuals and identify subtle genetic structure, informing conservation goals with evolutionary implications.

## Background

Caribbean coral reef communities have lost nearly 80 % of coral cover since the early 1980s [1] due to multiple interacting factors such as overfishing, eutrophication, climate change, storm damage, grazer die-off, and disease [2, 3]. Amongst Caribbean corals, the genus *Acropora* has experienced particularly large declines over the last 30 years, with losses exceeding 95 % in some areas [4] and up to 90 % region-wide [5], a decline unparalleled in the fossil record [6]. The staghorn coral *Acropora cervicornis* is the fastest growing Caribbean coral [7] and is thought to reproduce largely by fragmentation [8]. Thus, active restoration propagates coral fragments in nurseries prior to outplanting to depleted reefs and is an effective coral restoration technique [9]. Active restoration is especially important for reef-building corals that provide the bulk of the three-dimensional complexity on reefs and support critical ecological functions for many other reef-associated species. Restoration efforts must consider how corals will respond to changing environments in today’s oceans, where organisms may rely on a variety of responses, including physiological acclimatization or evolutionary adaptation [10–13]. With the increase in the number and scope of reef and coral restoration programs around the world, detailed knowledge is needed concerning the role that genetic diversity can play in the survivorship or remaining coral populations and the re-establishment of depleted populations based on nursery propagation.

Recent bottlenecks in the abundance of *A. cervicornis* can negatively impact this species’ genetic diversity. Reduced populations may lose uniquely adapted individuals and rare alleles, each important for adaptation and potential recovery. Reduced genetic diversity also can compromise successful sexual reproduction by decreasing the potential of cross-fertilization (acroporids have low self-fertilization success [14]). Since *A. cervicornis* appears to undergo limited sexual recruitment, in part due to spatial gaps between existing populations, enhancing densities using nursery-reared coral colonies has become a focal point for increasing the chances of a successful mass-spawning event [9]. To repopulate reefs and increase population densities, greater knowledge on the genetic structure of *A. cervicornis* is needed so management strategies can be tailored to the appropriate areas and spatial scales. Specifically since the potential for evolutionary adaptation is related to genetic diversity and is critical for the survivorship of any species in today’s changing environments [10], evaluation of genetic variation is needed to help recognize potential evolutionary outcomes and management repercussions.

Genetic variation in *A. cervicornis* shows significant regional structure (e.g.*,* between Florida and the Bahamas) for populations separated by more than 500 km in both nuclear and mitochondrial genes, suggesting restricted gene flow over large distances and potentially isolated populations [15]. Yet, within a smaller region like the Florida Reef Tract (FRT), analysis of *A. cervicornis* using microsatellites showed little population differentiation and no significant population structure [16]; these results were confirmed with mitochondrial control region sequences that showed no significant population structure for staghorn corals within the FRT based on data from 52 individuals [17]. Although most genetic diversity is related to large distances among regions, population structure was detected over smaller spatial scales (as small as 2 km) in 3 of the 20 areas examined [15]. This rare, fine scale structure was attributed to one-way introgression of *A. palmata* into *A. cervicornis* [15]. The finding of moderate genetic structure among regions in the Caribbean separated by more than 500 km suggests that these distant areas require independent conservation and management practices. Approaches that provide higher differentiation at smaller scales would highlight the need for more local management and restoration strategies. These approaches require new techniques to resolve any meaningful genetic variation.

**Fig. 1:**
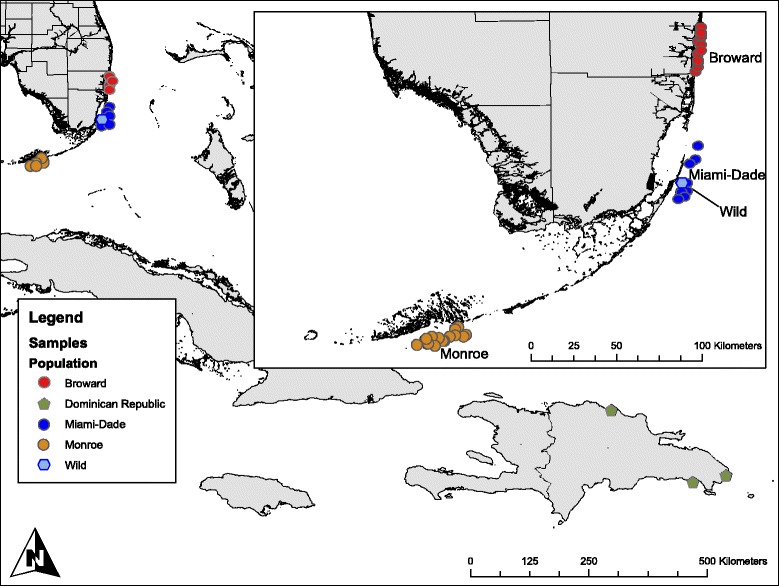
Locations of coral collection sites in Broward, Miami-Dade, and Monroe counties Florida and Dominican Republic. Wild was a single coral reef where all ten individuals were collected >50 m apart

Recently, the ability to quantify genetic variation has greatly improved with the use of next-generation sequence technologies [18]. It is now possible to genotype large numbers of individuals at thousands of loci using Genotyping by Sequencing (GBS) [19]. Here we use GBS to investigate the genetic diversity within and among *A. cervicornis* populations using individuals collected throughout the FRT) with individuals from the Dominican Republic used as an outgroup (Fig. 1; map was drawn using ESRI ArcMap 10.2). All individuals (except “Wild”) are harbored in a network of in-situ nurseries, which represent critical repositories of genetic data [20] and the sustainable source of coral tissue being used for active restoration of this threatened species.

## Methods

### Coral collections

A total of 77 samples were collected and analyzed: 66 individuals along the Florida Reef Tract and 11 individuals from around the Dominican Republic (Table 1). Dominican Republic corals were collected to serve as an out-group, enabling comparisons within the FRT to be considered relative to regional differences. Fifty-six FRT samples were received directly from a network of *in situ* nurseries harboring multiple corals originally collected from at least 500 m apart in separate reefs and tracked during propagation for active restoration efforts. Each of the FRT nursery corals was a unique genotype [21], which could potentially lead to an over-estimation of genetic diversity. An additional ten samples were collected from a single Miami-Dade county reef termed ‘Wild’ at 10-50 m intervals for a total of 66 colonies (Table 1) from 57 sites along the FRT. Nursery collections were used because they represent past wide sampling effort, making current collection efforts more efficient. Samples were considered to be *A. cervicornis* based on morphology and microsatellite tags, with the exception of the ‘Wild’ site, which was determined solely based on morphology. Other studies have discovered significant one-way introgression between *A. palmata* and *A. cervicornis* [15]; however the role of introgression is beyond the scope of the present study as the use of several thousand loci precludes the ability to compare individual genes to known *A. palmata* sequences. All corals were sampled by slicing a ~0.5 cm apical tip with a clean razor blade and placing the tip in 320uL of a chaotropic salt solution while in the field (4.5 M guanadinium thiocynate, 2 % N-lauroylsarcosine, 50 mM EDTA, 25 mM Tris–HCl pH 7.5, 0.2 % antifoam, 0.1 M β-mercaptoethanol) [22]. Samples were transported back to the University of Miami/RSMAS and stored at 4 °C prior to processing.

**Table 1:** Sample collection locations for the 77 samples analyzed

Region	Population	Samples Analyzed
Florida	Broward (NSU)	23
	Miami-Dade (UM)	10
	Wild (Miami-Dade)	10
	Monroe (MOTE)	23
Dom. Republic	Punta Cana (PCEF)	11
Total		77

Parentheses indicate nursery management institution: Nova Southeastern University, University of Miami – Rosenstiel School of Marine and Atmospheric Science, Mote Marine Lab, Punta Cana Ecological Foundation

Collections were made under the following permits: Convention on International Trade in Endangered Species of Wild Fauna and Flora Permits 11US835702/9, United States Department of the Interior National Park Service Scientific Research and Collecting Permits BISC-2013-SCI-0010, NOAA Florida Keys National Marine Sanctuary Research Permit FKNMS-2011-150, and Florida Fish and Wildlife Conservation Commission Special Activity License SAL-13-1086-SCRP.

### Genomic DNA and GBS

Genomic DNA was isolated using a silica column as described in [23]. Isolated DNA quality was assessed via gel electrophoresis and concentrations were quantified using Biotium AccuBlueTM High Sensitivity dsDNA Quantitative Solution according to manufacturer’s instructions. After quantification, 100 ng of DNA from each sample was dried down in a 96-well plate. Samples were then hydrated overnight with 5 ul of water before restriction enzyme digestion and further processing. GBS was preformed using the restriction enzyme ApeKI, unique barcoded adapters (0.4pmol/sample) and 50 ng of genomic DNA as described in [19]. A range of PCR cycles was used to optimize the amplification of restriction fragments using primers that anneal to the adapters. DNA from the 18-cycle run was pooled, and the GBS library was sequenced (Illumina Hi Seq 2500, 100 bp single end reads; Elim Biopharmaceuticals, Inc., Hayward, CA).

### Data processing and analysis

Raw Illumina sequences were received from Elim Biopharmaceuticals and processed using the GBS analysis pipeline TASSEL 4.0 [24]. The TASSEL pipeline trims sequence reads to 64 bp and removes reads that do not contain a cut site and barcode (to remove barcode dimer sequences); reads that did not meet these requirements were discarded. Reads were then aligned to the *A. digitifera* genome (the only published acroporid genome) to prevent the inclusion of *Symbiodinium* DNA, which would be present in background levels in any coral sample. Aligning to a genome also enhances the identification of allelic SNPs at a specific locus because sequence reads that match two or more locations in the genome are discarded [24]. This selection of SNPs that align to one location is only possible with a reference genome. Alignment to the *A. digitifera* genome results in unique sequence tags, which are aligned, 64 bp sequence reads that have a unique genome location. The TASSEL pipeline with BWA and Bowtie was used to call SNPs with a minimum allele frequency of at least 5 % and a minimum of 5 reads per locus to reduce the impact of sequencing error (by ensuring minimum frequency and number of reads the likelihood of false polymorphism calls decreases). Only loci called by both alignment tools were used to produce a conservative selection of loci for analysis. Before downstream processing, SNPs were filtered using an iterative progression to select individuals with at least 70 % of the called loci and loci that were present in at least 90 % of samples for analyses. Arlequin v.3.11 [25] was used to test Hardy-Weinberg Equilibrium and calculate genetic diversity among coral collections by calculating the percentage of polymorphic SNPs, observed heterozygosity (H_O_), expected heterozygosity (H_E_) and fixation index (F_ST_). Loci with significantly greater observed than expected heterozygosity (*p* < 0.01) were discarded from analysis, and loci with significant linkage-disequilibrium (D’ *p*-value < 0.01, or an r^2^ > 0.20) were identified using Tassel [24] with a 100 SNP sliding window (where the order of SNPs are defined by the *A. digitifera* genome) and removed.

For comparisons within populations, π, pairwise differences (different SNPs between samples/total SNPs*100), was calculated using the ‘ape’ package in R [26]. SNP π values were compared to more traditional measures of DNA sequence variation by correcting for the number of non-variable sites within each 64 bp sequence tag. Specifically, there are, on average, 1.4 SNP per 64 bp per sequence tag. Thus the average π * (1.4 SNP/64 bp sequence tag) provides an estimate of π when comparing DNA sequences with both invariable and polymorphic sites. For comparisons between populations, fixation index deviations from zero were tested by 10,000 permutations of alleles between individuals. To identify SNPs with F_ST_ outlier values (values larger than expected based on the observed data, [27]), the program LOSITAN [28] was used to generate 100,000 simulated SNPs, providing an expected neutral distribution of F_ST_ values and an estimate of P-values for each SNP. Structure [27] was used to identify the number of groups with similar allele frequencies (K). A model allowing admixture and correlated gene frequencies was used to carry out a total of 49 runs with seven independent runs for each K-value from 1–7. Ten-thousand permutations with 11,121 initial runs (burn-in) was used for each run. The K with the largest rate of change in the probability between groups was used to select the most parsimonious cluster [28]. RaXML was used to build a maximum likelihood tree [29] with 100 rapid bootstrap inferences. The best maximum likelihood tree (using a general time reversible model of nucleotide substitution and the Γ model of rate heterogeneity with ascertainment bias correction [30]) was selected and visualized using Dendroscope [31]. Discrimination analysis and comparisons of genetic and geographic distances (Mantel Test) were completed using ‘adegenet’ package in R [32, 33]. The Mantel Test was completed using a matrix of pair-wise differences in allele frequencies (Euclidean distances) and a matrix of geographic distances calculated from collection coordinates. Discrimination analyses (DAPC) was conducted in R using ‘adegenet’ [34]. DAPC was used in addition to Structure because it provides another metric of population differentiation, which does not assume un-relatedness, so potentially closely related individuals may be included.

## Results and discussion

### Coral samples

Table 1 lists the sample size for the 5 collections (Fig. 1). Along the Florida Reef Track, 56 individuals were sampled from separate reefs in Broward (Brwd), Miami-Dade (MD), and Monroe (Monr) Counties. We treated each county as a population. In addition to these three collections, 10 individuals were sampled from a single small reef in Miami-Dade (Wild), and 11 individuals were sampled from 11 locations in the Dominican Republic. Both Wild and the Dominican Republic were treated as separate populations. In previous studies [14, 15, 35] the FRT and DR would have been considered different regions, and the locations along the FRT were considered populations. In this study, the only difference is our treatment of the single reef with ten individuals sampled as a separate population (Wild). Although technically Wild is within Miami-Dade, the 10 individuals treated as a separate population provide insight not possible if merged with the 10 other individuals from separate reefs.

### GBS samples and sequencing

Next-generation sequencing and identification of informative SNPs requires: i) filtering the data such that most SNPs occur in most individuals, ii) removing inappropriate SNPs that represent nucleotide differences between paralogs (different loci) *versus* polymorphisms between alleles and, iii) eliminating SNPs in linkage-disequilibrium [18, 19, 36–40]. The results of this filtering are shown and discussed below.

Sequencing data returned a total of 159,634,510 sequences and 91,643,894 (57.4 %) were retained because they contained both the barcode and cut site. Then, these retained sequences were aligned to the *A. digitifera* genome [41]. Alignment to the *A. digitifera* genome was used to remove *Symbiodinium* sequences. In total, 868,023 unique sequence tags (tags are aligned, 64 bp sequence reads that have a unique genome location) aligned to the published reference genome [41]. Sequence tags removed due to lack of alignment with the *A. digitifera* genome were not analyzed because of potential background symbiont or bacterial DNA contamination. After preliminary filtering, three individuals were discarded because they had less than 30 % of the sequence reads. It is possible that inefficiencies during library construction led to low numbers of reads in these individuals. The remaining 77 individuals had 400,000 to 2,300,000 reads per individual.

Short 64 bp sequences were aligned using two alignment tools, Bowtie [42] and BWA [43], to identify Single Nucleotide Polymorphisms (SNPs). Bowtie identified 306,643 SNPs and BWA identified 178,644 SNPs, of which 113,838 SNPs were called by both alignment tools. These 113,838 SNPs were iteratively filtered to meet two criteria: individuals with 70 % of all called SNPs and loci that were present in 90 % of individuals. These criteria produced a total of 5,230 SNPs. These differences in alignment tools affect allele frequency, and for these data, there was a substantial difference in the observed heterozygosity: BWA identified many more loci with large heterozygosity values. Only approximately 50 % of reads shared alignments with these two tools, which has been observed in other studies [44–46]. Taking a conservative approach to avoid errors due to alignment tools, only the 5,230 SNPs identified by both alignment tools with similar or identical allele frequencies were considered. An additional 466 SNPs were removed because of excessive observed heterozygosity or linkage to another SNP, leaving 4,764 SNPs. SNPs that had observed heterozygosity significantly greater than expected (i.e., not in Hardy-Weinberg equilibrium) were removed because they most likely represent alignments between different loci and not real polymorphisms at the same locus [47]. Eliminating these SNPs with excessive observed heterozygosity was done to reduce the technical error caused by mis-alignment. However this also could eliminate loci strongly affected by balancing selection. Thus, we err on the side of reduced technical error with the potential loss of SNPs affected by balancing selection. We also removed one of each pair of SNPs with significant linkage disequilibrium (D’ *p*-value < 0.01, or an r^2^ > 0.20) [48]. Removing SNPs in linkage-disequilibrium (LD) should not bias the measure of variance unless SNPs in LD have significantly greater or less variation. Strong LD is associated with background selection or directional selection, both of which would reduce variation. Thus, our SNP measurements may be conservative estimates of the variation present. After removing loci not in HWE and loci with significant linkage disequilibrium, 4,764 SNPs remained. These 4,764 SNPs were used for all analyses.

There is an additional concern that *Symbiodinium* may have conserved genes that could align to the *A. digitifera* genome. This would seem unlikely because if there were multiple types of *Symbiodinium,* these should have excessive observed heterozygosity and would be filtered out. Yet, to examine this possibility we used BLAST-N with all sequence tags that define the 4,764 SNP against the *Symbiodinium minutum* Mf 1.05b.01 (taxid:1280413) genome. Only one tag had an e-value < 10^−5^, and this sequence only matched 83 % of the genome for 49 of the 64 bp (63 % similarity for all 64 bp). Eight more tags had e-values < 1 %, but none of these matched more than half of the sequence tag. Thus, it seems unlikely that the alignments contain *Symbiodinium* sequences.

### Genetic diversity within populations

The genetic diversity within a population can be represented by π, the nucleotide diversity or number of nucleotide differences among pairs of samples divided by the number of SNPs. Here, these data are represented as pairwise differences (π,) between samples. The π within each FRT population (Broward, Miami-Dade, Wild, and Monroe) averages 37 % with a range of 23.9–44.0 % (Table 2). This indicates that among all SNPs, an average of 37 % are different between any two individuals within a single population. The differences within a population are similar to the overall value when all 5 populations (including DR samples) are used. While only the ‘Wild’ site represented multiple individual colonies sampled from a single reef, π in Wild remained high (average 37 %, range 24.9 to 43.8 %) and in the range of π in the other populations (23.5 % to 44.0 %). Among the three FRT transects Broward and Monroe had an average π of 38.3 % and 39.5 %, respectively, but narrower range of π (35 % to 41.2 %, 36.2 to 43.8 %) than Miami-Dade. This was somewhat surprising because *A. cervicornis* in Broward County is growing at the edge of its spatial range, so high diversity may be unexpected. Importantly, within each population π measurements are comparable to π across populations (Table 3), indicating that genetic diversity starts at the local level, including single reefs. The measures presented here represent large enough differences between individuals that each individual collected represents a unique individual (minimum π: 23.5 %), including those from the single reef (‘Wild’).

**Table 2:** Nucleotide diversity or pairwise differences (π) among individuals within a population

π, Pairwise differences			
	Avg.	Min.	Max.
All Pops	0.392	0.235	0.440
Broward	0.383	0.350	0.412
Miami-Dade	0.383	0.235	0.416
Monroe	0.395	0.362	0.435
Wild	0.370	0.249	0.438
Dom. Republic	0.363	0.267	0.440

Average among all individuals and minimum and maximum differences for a pair of individuals within a population

**Table 3:** Pairwise differences (π) between populations

	Broward	Miami-Dade	Monroe	Wild	Dom. Republic
Broward	0.383				
Miami-Dade	0.389	0.383			
Monroe	0.392	0.393	0.395		
Wild	0.392	0.395	0.398	0.370	
Dom. Republic	0.391	0.398	0.402	0.400	0.363

Average π among every individual from each population compared

Traditionally, *A. cervicornis* is thought to rely primarily on asexual propagation, so single reefs have been believed to be monotypic or have few genets [7, 49]. The observed level of diversity between individuals is also unexpected because previous genetic analyses of *A. palmata* indicate a high ramet/genet ratio where many reefs may be populated by one or a few genets [50]. Even though the dataset presented here only examines polymorphic sites, the similar levels of genetic variation within and among populations indicates that much diversity occurs among individuals. Additional evidence suggests that genetic diversity is occurring over even smaller spatial scales, where multiple colonies in close proximity (<5 m apart) show similar π and are likely unique genets (Drury, unpublished data). This level of genetic diversity is unlikely to be an artifact. All SNPs were called in 90 % of all individuals with a minimum allele frequency of 5 %. There was an average of 411 reads per SNP and 69 reads per SNP for the minor allele. Furthermore, 90 % of the minor alleles had more than 23 reads while the minimum number of reads for all SNPs was 8. Thus it seems unlikely that the detected SNPs are sequencing errors. Importantly, these measures are conservative because the sample size per population (n = 10 to 23) may overlook minor alleles and 5 % minimum allele frequency will underestimate the genetic divergence. Additionally, removing SNPs in LD and with excessive heterozygosity could reduce these estimates of genetic variation. Thus, our data are indicative of large standing genetic variation within populations, but they may be under-estimates.

Measures of π within *A. cervicornis* populations are similar to values in other GBS studies on stickleback and natural populations of *Saccharomyces cerevisiae* [51, 52]. Yet to compare our GBS measures directly to more traditional π values for complete gene sequences requires the frequency of SNPs and invariable sites within each tag. This value is estimated by dividing number of SNPs on a given tag by the length of reads; for all pooled polymorphic tags investigated here, there were an average of 1.45 SNPS per 64 bp equating to an adjusted π value of 0.9 %. This adjusted π value is similar to nucleotide diversity across Caribbean populations for the three nuclear genes in *A. cervicornis* [15], but nearly four times as large as π among Florida populations, estimated as 0.002 using the mitochondrial control region [17]. When compared across a wide array of taxa, π = 0.9 % is a substantial level of genetic variation relative to most animals [53].

The 57 individuals from the Florida nursery collections (Broward, Miami-Dade and Monroe, but not Wild, Table 1) were identified as unique genotypes by microsatellites within each population (Baums, unpublished data). Among these individuals there was an average π of 39.1 % (23.5 % to 43.5 %). Each of these samples was a different individual with the minimum π of 23.5 % between individuals (i.e., two individual were different at 23.5 % of SNPs). This non-random collection could inflate π within each of these three populations but should not inflate the variation among populations. Yet, π among these 57 individuals is similar to that of the single reef collection (Wild) and is similar to π among all populations (Table 2). Thus, within each of the three Florida transects, which were separate nursery collections, π was similar to that of a single reef and did not exceed π between individuals in different transects. This suggests that π is not greatly inflated by the selection of individuals with different microsatellite genotype. Although there may be a slight bias in π for the three FRT nurseries, our data indicate large, standing genetic variation among FRT corals and genetic variation within *A. cervicornis* populations along the FRT similar to the variation across all populations. The amount of variation discovered within and among *A. cervicornis* populations is similar to the genetic variation found in large outbred populations distributed over large geographic ranges [51–53]. Despite the dramatic decline in the census population, this decline did not result in substantial loss of genetic diversity, suggesting that remaining corals are i) old individuals sampled from a population with large diversity and a very large effective population size, ii) new recruits from a wide variety of parental inputs, or iii) are affected by non-neutral processes enhancing genetic diversity (*e.g.,* divergent selection on different habitats/reefs).

### Genetic diversity among populations

**Fig. 2:**
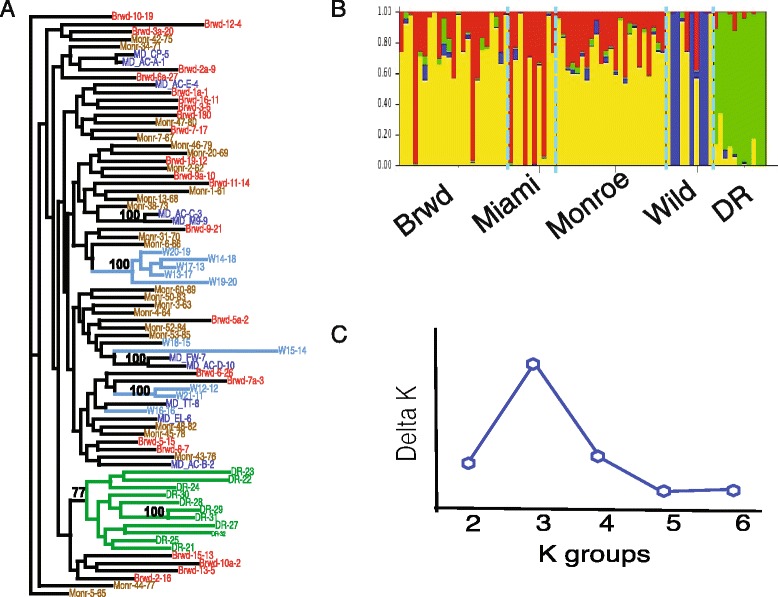
Maximum likelihood tree and Structure showing shared relationships among individuals based on 4.7 K SNP. **a** Maximum-likelihood tree with 100 bootstraps; only branches with >70 % support are enumerated. Taxa are color-coded (Broward: red, Miami-Dade: dark blue, Monroe: brown, Wild: light-blue and Dominican Republic: green). Clades for Wild have blue branches and the clade for Dominican Republic has green branches (**b**) Structure plot for five populations. Each individual is labeled with the color related to the predicted population. **c** Delta K; rate of change in the log probability of data between successive K values [28]

Substantial genetic diversity within populations is accompanied by significant divergence among populations and is illustrated by the relationships among individuals seen in the maximum-likelihood tree and Structure Plot (Fig. 2a, b). The maximum likelihood tree using 4,764 SNPs has six branches with over 70 % bootstrap support, including all individuals from the Dominican Republic in a single cluster with 100 % support on internal branches. Other branches with 100 % bootstrap support include pairs of individuals from Miami-Dade and two sets of colonies from the wild reef (with two and five individuals respectively) (Fig. 2a). Structure analysis suggests 3 or 4 groups of individuals have similar minimum mean likelihood Fig. 2b). Each column (Fig. 2b) represents an individual and the summary of allele frequencies for that individual. Based on rate-of-change likelihood, separation into 3 groups is the most parsimonious explanation for the data (Fig. 2c), with Broward, Miami-Dade, and Monroe populations sharing common allele frequencies while each is divergent from the Wild population and the Dominican Republic samples. Regional differences, i.e., Florida *vs*. Dominican Republic, agree with previous reports of Caribbean-scale population structure in *A. cervicornis* [16, 17]. Collections from the ‘Wild’ reef appear to have 50 % of individuals with divergent ancestry from other FRT (Fig. 2b) and share a well supported clade in the maximum-likelihood tree (Fig. 2a). The phylogeny and structure plots indicate differences within the FRT as a whole, but do not readily resolve differences within the three sub-regions.

To further resolve differences among populations, we applied four different analyses: 1) a hierarchical AMOVA [54], 2) analysis of F_ST_ values across all loci, for individual loci and for outlier F_ST_ values [55], 3) a Mantel test and 4) discrimination analysis of principal components (DAPC).

An AMOVA of all 4,764 loci for two groups (Florida and DR) with four populations within Florida (Broward, Miami-Dade, Monroe and Wild) shows significant variation (*p* < 0.001) among groups, among populations within Florida and within populations (Table 4). The significant difference among groups confirms the regional differences between the Dominican Republic and Florida seen in the Structure analyses and maximum likelihood tree (Fig. 2). Greater than 90 % of the observed variance is within populations (Table 4), but there are also significant differences among the FRT nursery populations accounting for approximately 2 % of the variation. The genome-wide F_ST_ values (Table 5) are significant for all but Monroe and Broward pairwise comparisons and represent the first genetic structure formally resolved in Florida *A. cervicornis*. These F_ST_ values are not large (range: 0.016 to 0.092), so they may be more reflective of the statistical power of using many loci [56] and less ecologically relevant; nevertheless they represent a novel ability to distinguish between sub-regions of the Florida Reef Tract, which has been viewed as relatively homogenous in previous investigations [16, 17]. Interestingly, among the three Florida transects, Miami-Dade exhibits significant divergence in comparison to Monroe (~180 km) and Broward (~60 km) samples, but the latter transects are not significantly different despite larger spatial separation (~250 km).

**Table 4:** AMOVA design and results based on 4,764 SNPs

Source of variation	Sum of Squares	Variance components	Percent variation
Among Groups	1916.1	37.685	4.35
Among Pops within Groups	3101.2	627.813	2.16
Within Populations	85576.0	664.813	93.49
Total	90593.3	664.813	

Groups are FL and DR, with four populations within FL (Broward, Miami-Dade, Monroe and Wild)

**Table 5:** F_ST_ and *P*-values

	Broward	Miami-Dade	Monroe	Wild	Dom. Republic
Broward	------	0.009	0.839	0.000	0.000
Miami-Dade	0.016	------	0.000	0.009	0.000
Monroe	0.003	0.012	------	0.000	0.000
Wild	0.041	0.045	0.034	------	0.000
Dom. Republic	0.056	0.069	0.050	0.092	------

F_ST_ values are based on all 4.7 K SNP. Below the diagonal are the F_ST_ values. Above the diagonal are the *p*-values for the specific comparisons; all comparisons are significant except Broward *vs.* Monroe

**Fig. 3:**
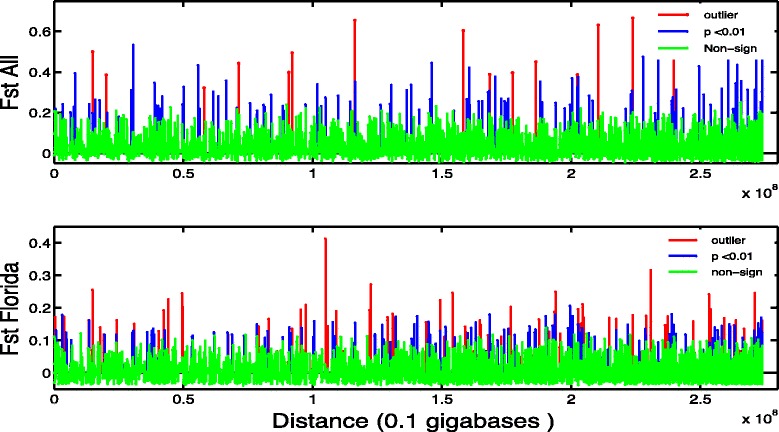
Locus specific genetic distance (F_ST_ value) by position relative to the *A. digitifera* genome. Significant F_ST_ values that are blue, and the subset that are outliers (potentially adaptive) are in red. SNPs with non-significant F_ST_ values are green. Distance along the X-axis is the sum of distances among the 4,765 scaffolds. **a** F_ST_ values for all five populations. **b** F_ST_ values for only the three Florida transects (i.e., Broward, Miami-Dade and Monroe, without Wild)

Among the three Florida transects (i.e., Broward, Miami-Dade and Monroe, without Wild), there are 300 SNPs with significant F_ST_ values (*p* < 0.01; Fig. 3, Table 6). Most SNPs with significant F_ST_ values among FRT transects are not physically close to each other (average distance is 88,235 bp), suggesting that most SNPs are evolving independently and the differences among populations are not due to one or a few linked loci. The average distance to the next closest SNP is > 25 kb (although many non-significant SNPs are often on the same 64 bp sequence tag). These close, non-significant SNPs indicate lack of linkage or selective sweeps and high, long-term standing genetic variation. The individuals sampled from the Miami-Dade nursery should have captured much of the local genetic variation as they were initially collected from a large area (~35 km span), collections were separated by at least 500 m, and all represent unique genets [57].

**Table 6:** F_ST_ values and nucleotide distance for all five populations and three Florida transects

	F_ST_ all SNP	Significant F_ST_	Outlier F_ST_	Next non-significant F_ST_	Distance (bp) among Significant F_ST_ SNP	Distance (bp) among Outlier F_ST_ SNP	Distance (bp) between significant and non-significant SNP
	All 5 populations						
Count^a^	4,762	141	17	108	23	0	108
Average	0.040	0.3018	0.4454	0.0989	75,876	N/A	31,576
95 % CI^b^	0.0383, 0.0424	0.2835, 0.3200	0.3607, 0.5300	0.0780, 0.1198	2,338, 14,9414	N/A	22,578, 40,573
Read^c^ (range)	411 (125:4,142)	377 (198:1,097)	367 (256:679)	405 (251:841)			
	3 Florida Populations (Broward, Miami-Dade, and Monroe)						
Count^a^	4,753	300	150	207	73	28	207
Average	0.012	0.1163	0.1165	0.0451	88,235	46,304	27,698
95 % CI^b^	0.0099, 0.014	0.1097, 0.1229	0.1046, 0.1283	0.0363, 0.0539	55,264, 12,1207	4,627, 97,235	20,523, 34,872
Reads^c^ (range)	411 (125:4,142)	370 (125:967)	377 (167:967)	402 (208:947)			

^a^Counts refer to the number of polymorphic SNPs used in the analyses or (for distance) the pair of SNPs that shared the same scaffold. ^b^CI is the 95 % confidence interval. ^c^Reads are the average number of 64 bp reads for each SNP. “Significant” and “non-siginficant” refers to SNPs with statistically significant F_ST_ values

To further parse differences between sub-regions, a Mantel Test was used to calculate the correlation between genetic and geographic distance. Here, 377 SNPs with significant F_ST_ values (from locus-specific F_ST_ values, Fig. 3) were compared to geographic coordinates from the original collection sites. Among all populations, there is a significant (*p* < 0.001) correlation between genetic variation and spatial distribution; this trend explains approximately 38 % of the genetic variation (R^2^ = 0.378) and is driven mainly by the differences between Florida and the Dominican Republic, supporting the regional structure previously reported [15]. When examining only the corals from the three Florida transects, the relationship is significant but explains much less variation (R^2^ = 0.104). Importantly, the genetic variation explained by geographic separation increases if either Broward or Monroe (the northernmost and southernmost Florida sub-regions, respectively) is excluded, because these two most distant sub-regions of the FRT are more similar to each other than either is to the spatially intermediate Miami-Dade corals. The results of the Mantel test support the pairwise F_ST_ values, which indicate significant structure between Miami-Dade and Monroe/Broward, but little genetic divergence between Monroe and Broward.

**Fig. 4:**
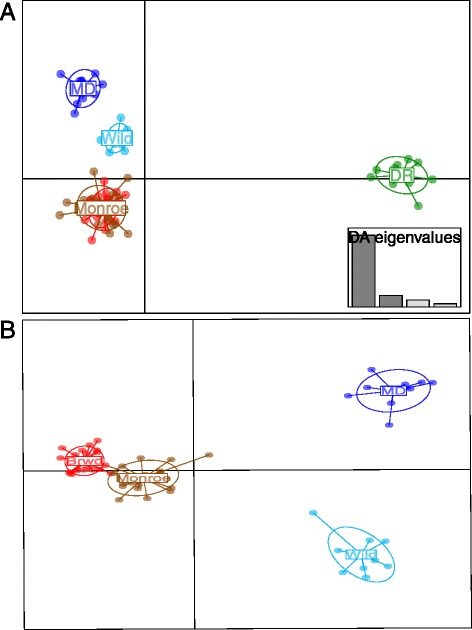
Discriminant analysis of populations. Discriminant analysis of principal components [34] was used to define the similarity and differences for (**a**) all five populations and (**b**) the four Florida populations. Populations are shown by different colors and inertia ellipses while dots represent individuals: Broward: Brwd-red, Miami-Dade: MD, dark blue, Monroe: brown and Dominican Republic: DR, green

Using DAPC with 4,764 SNPs shows that the first discriminant function separates the Dominican Republic from the Florida corals, while the second discriminant function separates the four Florida populations (Fig. 4a). When only the four Florida populations are analyzed (Fig. 4b), there is clear discrimination among three of the four populations, with little difference between the Monroe and Broward (Fig. 4b), supporting conclusions in the Structure and AMOVA analyses. Although the Miami-Dade and ‘Wild’ individuals were collected from the same area, they are more readily distinguished from each other in comparison to the two most distant collections (Broward and Monroe). Despite significant F_ST_ value differences between the Miami-Dade nursery and Miami-Dade ‘Wild’ collections, π is similar in both collections (38.3 % *vs*. 37.0 %, respectively), suggesting a change in allele frequencies associated with the local environment. These data suggest there are many genetic differences among populations and genetic diversity is high in each of the three Florida transects. These data also support the conclusion that the differences among collections are not a linear function of geographic distance within Florida; geographically close collections may have more differences that geographically dispersed collections.

### Adaptive genes

Corals from the three Florida transects had 300 loci with significant F_ST_ values. Half or 150 of these SNPs were outliers. Outlier SNPs have F_ST_ values with changes in allele frequencies that are not found in 100,000 random permutations of the data and are thought to be due adaptive evolution [58, 59]. Although outlier tests suffer from both type I and II errors [60], a stepping-stone model of divergence is likely to be similar to the connectivity of the FRT populations, and thus the outlier test we used is unlikely to suffer from extensive type I errors [60]. Thus we conclude that some of the divergence among the FRT populations likely reflects adaptive evolution.

It has not escaped our attention that the Wild collection of 10 individuals within a single reef within Miami-Dade is different from the Miami-Dade transect collected from the surrounding area. While these individuals from Miami-Dade and Wild are phylogenetically similar to the other samples (they share internal branches in the phylogenetic tree), the comparison between these two populations shows 260 SNPs with significant F_ST_ values (average F_ST_ value = 0.232, range: 0.169 to 0.824). Each of these SNPs has an average of 120 reads among the twenty individuals from Miami-Dade and Wild populations (range: 45 to 1,048) and over 350 reads among all individuals. Two-hundred and thirty-four (89 %) of these SNPs with significant F_ST_ values are significant outliers [55]. The divergence between Wild and the Miami-Dade transect could represent local adaptation or could arise if the Wild individuals contained *A. cervicornis – A. palmata* hybrids. Unlike the other collections, which were identified based on morphology and microsatellite tags, the Wild collection was only identified by morphology. Although we have no reason to believe that the Wild samples were hybrids, the fine scale genetic divergence we found is similar to the rare, fine scale structure that was attributed to one-way introgression of *A. palmata* into *A. cervicornis* [15]. Thus although it is intriguing that natural selection is acting on a fine geographic scale, this conclusion may be premature until the species status of the Wild population is investigated.

### Implications for restoration

Data presented here suggest that there is potentially much adaptive variation due to subtle environmental differences influencing coral distribution and growth, including temperature, water chemistry, light, nutrients, and sedimentation. This variation may occur over spatial scales as small as individual reefs. Due to the potential for high adaptive variation, introducing a broad range of genotypes along the FRT (such as those housed within nurseries) would enhance the frequency of adaptive genotypes and the subsequent rate of offspring survival. This is especially true if crossings during mass spawning events produce a larger range of genotypes able to take advantage of a large breadth of ecological niches. Thus, the best conservation and restoration strategy may be to increase genetic variation on all spatial scales (within reefs, among populations) as much as possible to provide diversity to cope with changing conditions [10].

This study found substantial genetic variation within existing staghorn populations being raised in coral nurseries. These nursery corals are presently used for coral propagation and outplanted to enhance population recovery of the threatened staghorn coral reefs. Greater than 90 % of the variation among all the samples is found within a nursery’s collection of corals, indicating that these nurseries have captured significant genetic diversity.

These GBS data indicate both large variation within populations and adaptive divergence among populations, and should help form policies that guide conservation efforts to restore staghorn coral reefs. We suggest that the caution against moving corals long distances during restoration [61] should be tempered, because genetic variation is very high within single reefs and among the three populations along Florida transects. Previous consideration of the implications of redistribution of corals during restoration suggests that moving corals beyond some ecologically relevant threshold may result in decreased fitness of a restored population due to founder effects, genetic swamping and inbreeding/outbreeding depression [61]. Here, we argue that the very high levels of diversity found within nursery source materials and on a single reef alleviate some concern. Very diverse assemblages on reefs targeted for restoration and in nursery source corals will likely not undergo fitness declines due to genetic swamping or outbreeding depression because there is much genetic variation within populations and no unique alleles in any of the Florida populations. Although there may be some reefs with one or few remaining colonies that have unique adaptive alleles, introducing genetically diverse corals would increase the genetic variation of any resulting coral larvae, and this diversity is needed for adaptation. Similarly, the potential for significant inbreeding depression would be decreased by the introduction of diverse coral assemblages. Outbreeding depression remains a concern. Yet in extant coral reefs, large genetic variation occurs within and among reefs. Thus, concerns about outbreeding depression for sexually produced coral larvae that will disperse long distances and face changing environments seems misplaced.

## Conclusions

The GBS approach produced genotype frequencies for 4,764 SNPs that allowed for the resolution of population differences unavailable using other techniques [15–17]. Each SNP had an average of 411 reads/SNP with 69 reads/SNP for the minor allele, so genetic differences likely represent real nucleotide divergence and not sequencing error. However, there are imperfections to this approach, including the differences in heterozygosity produced by different SNP alignment tools. Despite the caveats with this method, GBS provides the ability to resolve previously undiscovered variation in populations of *A. cervicornis.* Here, we show, for the first time, population structure across the FRT and high diversity within populations, including within a single reef evidenced as the genetic structure between and among FRT populations. Previous work on *A. cervicornis* using mtDNA, a few nuclear genes, and microsatellites found no difference among the FRT coral populations [15–17, 62].

To further develop effective conservation and management plans for this species and other threatened corals considered as candidates for active propagation and restoration, it is essential to understand the extent of genetic variation within and among populations [15, 17]. Using a GBS approach, we highlight population differences by revealing many SNPs that have distinct allele frequencies among populations including one hundred and fifty SNPs, which have outlier F_ST_ values indicative of adaptive difference. There are also significant differences over small spatial scales, exemplified by differences between Wild and Miami-Dade individuals that were all collected within the same area (Fig. 1). The high genetic variation present in FRT *A. cervicornis* may allow this species to endure the interacting threats posed by local stressors and climate change factors such as temperature anomalies and acidification Additionally, π pair-wise differences) is large (37 %) for all collections and similar to GBS measures of π in large outbred populations of 3-spine stickleback or natural populations of yeast [51, 52].

The GBS methodology used here highlights the ability to discover subtle changes in populations by using thousands of loci and large numbers of individuals. Conservation genetics using these high throughput techniques provide a new lens for assessing management implications and population connectivity via important increases in resolution, but also in varied and specific genetic metrics such as population structure, nucleotide diversity, and loci that may be under selection. These data are particularly important to active restoration projects as they give a better understanding of population structure, how and where to relocate coral, and potential repercussions of active intervention. Furthermore, the ability to describe genetic diversity over local to regional distributions enables conservation practitioners to manage resources over appropriate scales, becoming more efficient and effective. GBS allows for increased restoration effectiveness through conservation genetics, while developing a more thorough understanding of threatened coral communities.

### Availability of supporting data

Raw sequences have been submitted to NCBI as a Sequence Read Archive (SRA). The *Acropora cervicornis* hapmap (DOI: 10.6070/H4FB50XX) and sequence tags (DOI: 10.6070/H49K4872) are available at LabArchives.com. Sequence files are available at NCBI's BioSample database, accessions SAMN03295587 - SAMN03295662.

## Abbreviations

FRT: Florida reef tract
GBS: Genotyping by sequencing
SNP: Single nucleotide polymorphism
DAPC: Discriminant analysis of principal components
